# Post COVID-19 and Long COVID Symptoms in Otorhinolaryngology—A Narrative Review †

**DOI:** 10.3390/jcm14020506

**Published:** 2025-01-14

**Authors:** Orlando Guntinas-Lichius, Thomas Bitter, Robert Takes, Victor H. F. Lee, Nabil F. Saba, Antti A. Mäkitie, Luiz P. Kowalski, Iain J. Nixon, Alfio Ferlito

**Affiliations:** 1 Department of Otorhinolaryngology, Jena University Hospital, 07747 Jena, Germany; thomas.bitter@med.uni-jena.de; 2 Department of Otolaryngology-Head and Neck Surgery, Radboud University Medical Center, 6525 GA Nijmegen, The Netherlands; robert.takes@radboudumc.nl; 3 Department of Clinical Oncology, Li Ka Shing Faculty of Medicine, The University of Hong Kong, Pok Fu Lam, Hong Kong; vhflee@hku.hk; 4 Clinical Oncology Center, The University of Hong Kong-Shenzhen Hospital, Shenzhen 518053, China; 5 Department of Hematology and Medical Oncology, Emory University, Atlanta, GA 30322, USA; nfsaba@emory.edu; 6 Research Program in Systems Oncology, Department of Otorhinolaryngology-Head and Neck Surgery, University of Helsinki and Helsinki University Hospital, FI-00029 HUS Helsinki, Finland; antti.makitie@helsinki.fi; 7 Department of Otorhinolaryngology, Head and Neck Surgery, A.C. Camargo Cancer Center, São Paulo 01509-010, Brazil; lp_kowalski@uol.com.br; 8 Department of Head and Neck Surgery, University of São Paulo Medical School, São Paulo 05403-000, Brazil; 9 Department of Otorhinolaryngology-Head and Neck Surgery, NHS Lothian, Edinburgh EH1 3EG, UK; iain.nixon1@nhs.net; 10 International Head and Neck Scientific Group, 35100 Padua, Italy; profalfioferlito@gmail.com

**Keywords:** COVID-19 pandemic, SARS-CoV-2, post COVID-19, long COVID, otolaryngology, otology, rhinology, laryngology, olfaction, taste

## Abstract

Post/Long COVID (syndrome) is defined as a condition with symptoms persisting for more than 12 weeks after the onset of SARS-CoV-2 infection that cannot be explained otherwise. The prevalence of self-reported otorhinolaryngological Post/Long COVID symptoms is high. The aim of this review was to analyze the current literature regarding the actual prevalence, knowledge of the etiopathology, and evidence-based treatment recommendations of otorhinolaryngology-related Post/Long COVID symptoms. A systematic literature search of articles published since 2019 in PubMed and ScienceDirect was performed and resulted in 108 articles. These were the basis for this review and formed a comprehensive series of consented therapy statements on the most important of otorhinolaryngology-related Post/Long COVID symptoms. Otorhinolaryngological symptoms did not appear isolated but as part of a multi-organ syndrome. Self-reported otorhinolaryngology-related Post/Long COVID symptoms were often not confirmed by objective testing. The confirmed prevalence estimated for anosmia, dysgeusia, cough, facial palsy, hoarseness/dysphonia, acute hearing loss, tinnitus, and vertigo/dizziness was about 4%, 2%, 4–19%, 0%, 17–20%, 8%, 20%, and 5–26%, respectively. There are manifold theoretical concepts of the etiopathology of different symptoms, but there is no clear evidence-based proof. This certainly contributes to the fact that there is no effective specific treatment option for any of the symptoms mentioned. Healthcare pathways must be established so that otorhinolaryngological Post/Long COVID symptoms can be recognized and evaluated and otorhinolaryngologists can provide counseling. This would also help to establish and selectively include patients in clinical trials investigating specific therapeutic concepts.

## 1. Introduction

Various long-term health consequences related to a coronavirus disease (COVID) caused by a SARS-CoV-2 virus infection have been observed, which are summarized under post-acute sequelae of acute SARS-CoV-2 infection or by the terms Post COVID-19 and Long COVID [1]. The estimated cumulative global incidence of Long COVID for 2023 was 409 million cases [2]. According to previous findings, it can be assumed that Post COVID-19 and Long COVID are not a uniform clinical condition, but they are rather various possible long-term health sequelae after a previous SARS-CoV-2 infection. These can affect different organ systems, cause different symptoms, and also have different mechanisms. Possible long-term health consequences include a variety of physical, cognitive, and psychological symptoms that have a negative impact on everyday functioning and quality of life. More than 200 symptoms have been identified [3]. Impairments either occur during the acute phase of the disease and persist in the long term or reappear weeks or months after the primary infection. Different symptoms—including those in the field of otorhinolaryngology—are reported, which can occur alone or in combination and can vary in severity and duration.

The mechanisms of Post COVID-19 and Long COVID have not yet been sufficiently clarified [4]. There is now evidence that chronic inflammation and the occlusion of small vessels, viral persistence [5,6], the activation of the Epstein–Barr virus [7], changes in the gut microbiome [8,9], and autoimmune processes are involved in the development of long-term health consequences [10,11].

COVID-19 is characterized by a wide spectrum of disease severity, ranging from asymptomatic or oligosymptomatic cases to severe and life-threatening forms [12]. As the coronavirus SARS-CoV-2 is a respiratory virus, many symptoms caused by SARS-CoV-2 infection are related to the involvement of the upper respiratory tract. Therefore, symptoms in the field of otorhinolaryngology are common [13]. In addition, the most pathognomonic symptoms, i.e., alteration in smell, nasal obstruction, sore throat, and cough, have been consistently described as early symptoms of the disease, especially in the virus variants in the early years of the pandemic.

The narrative review presented here aims to provide an overview of the current knowledge regarding which otorhinolaryngological symptoms associated with the acute phase of COVID-19 may persist, specifically those that could be classified as part of Post COVID-19 and Long COVID. At present, the literature reviewing persistent otorhinolaryngological symptoms as part of Post COVID-19 and Long COVID focusing on causality is sparse [14]. Knowledge of prevalence rates and the possible predictors of Post COVID-19 and Long COVID symptoms in the field of otorhinolaryngology will be discussed in detail.

## 2. Definition of Post COVID-19 and Long COVID

The difference between Post COVID-19 and Long COVID is defined in terms of time period. According to the guideline recommendations published by the British National Institute for Health and Care Excellence (NICE) at the end of 2020 [15], Long COVID is defined as health complaints that persist or reappear beyond the acute phase of a SARS-CoV-2 infection of 4 weeks. Post COVID-19 (syndrome) is defined as symptoms that persist for more than 12 weeks after the onset of SARS-CoV-2 infection and cannot be explained otherwise. Long COVID therefore includes both symptoms that persist after an acute COVID-19 illness 4 to 12 weeks after the onset of symptoms and Post COVID-19 syndrome [16]. The German S1 Long/Post COVID-19 guidelines also mention the worsening of pre-existing underlying diseases as another possible manifestation of Long COVID [17].

The World Health Organization (WHO) defines Post COVID-19 syndrome as health complaints that persist or recur at longer intervals (usually three months) following a SARS-CoV-2 infection over a longer period of time and cannot be explained in any other way [18,19]. Symptoms and health restrictions that persist for at least two months or occur recurrently and with varying severity and which are generally associated with impairments in everyday functioning are taken into account. When following these definitions, the prevalence of Post/Long COVID is about <0.1–2% [20,21]. Studies not strictly following these definitions or only considering unvaccinated patients reported a prevalence of 10–40% of Post/Long COVID patients [22]. The WHO definition of Post COVID-19 for children and adolescents also takes into account persistent, new, or recurring health complaints that persist for at least two months and are generally associated with functional limitations. Recently, the U.S. American National Academies of Sciences, Engineering, and Medicine (NASEM) published a 2024 NASEM Long COVID definition. According to NASEM, Long COVID is an infection-associated chronic condition that occurs after SARS-CoV-2 infection and is present for at least 3 months as a continuous, relapsing and remitting, or progressive disease state that affects one or more organ systems [23]. NASEM sees the terms Post COVID-19 and Long COVID synonymously. In contrast, the post-acute sequelae of a SARS-CoV-2 or ongoing symptomatic COVID-19 case are defined as those lasting for a period of 4–12 weeks.

In the present review, we evaluated the literature until August 2024, following an older definition other than that in the new NASEM guidelines. The present study still followed the previous definition: Post/Long COVID is defined as symptoms that persist for more than 12 weeks after the onset of SARS-CoV-2 infection and cannot be explained otherwise. Long COVID therefore includes both symptoms that persist after an acute COVID-19 illness 4 to 12 weeks after the onset of symptoms and Post/Long COVID. We did not differentiate the so-called post-acute sequelae from Post/Long COVID, as they have often not been taken into account in the analyzed literature (see next chapter).

## 3. Materials and Methods

### 3.1. Preconditions for Otorhinolaryngological Post COVID-19 and Long COVID Symptoms

Following the definition of Post COVID-19 and Long COVID, symptoms in the field of otorhinolaryngology can only occur if the patient had a previous SARS-CoV-2 infection (at best confirmed by a positive PCR test; strictly speaking, a PCR test is not required for diagnosis) in a relevant epidemiological setting. Second, the reported otorhinolaryngological symptoms were also normally symptoms that appeared during the acute phase of the previous SARS-CoV-2 infection. This refers in particular to the loss of smell. It was decided for the purpose of this review that if the patient did not have an olfactory disorder during the primary infection and such a symptom occurred months later, it would not be included as a Post or Long COVID symptom. Other otorhinolaryngological symptoms can be delayed in onset for weeks or months following what appears to be full recovery from acute infection. Vertigo/dizziness and also tinnitus are such examples. Finally, patients with Post COVID-19 or Long COVID do not only have otorhinolaryngological symptoms. Typically, otorhinolaryngological symptoms are part of a symptom complex, i.e., the patient has Post or Long COVID myalgia, fatigue, and also otorhinolaryngological symptoms.

Alterations in smell and taste were early pathognomonic symptoms during the COVID-19 pandemic at the onset of the infection, especially for the virus variants in 2020 and 2021. About two-thirds of patients with mild-to-moderate COVID-19 disease reported smell and taste alterations [24]. Also, other otorhinolaryngological symptoms were among the most common acute symptoms. Upper respiratory tract symptoms like nasal obstruction, rhinorrhea, sneezing, cough, ear fullness, sore throat, neck swelling, hoarseness, and dizziness occurred in about 70% of overall symptoms in patients with mild-to-moderate SARS-CoV-2 infection [25,26,27,28]. For many other reported otorhinolaryngological symptoms, including acute hearing loss, facial palsy, and tinnitus, a causal relationship was not proven with satisfactory medical evidence. It is reasonable to assume that many reported links are only coincidental [12]. Many poor-quality studies were published during the pandemic that propagated associations between COVID-19 and otorhinolaryngological symptoms without being able to prove this scientifically [29].

### 3.2. Determination of COVID-19-Associated Otorhinolaryngological Symptoms

Tirelli and Boscolo-Rizzo classified the literature on reported otorhinolaryngological symptoms based on scientific quality into definitive, probable, or possible otorhinolaryngological symptoms of COVID-19 [12]. Furthermore, some reported symptoms were classified as complications of other COVID-19-related symptoms (for instance, intubation injury-related symptoms due to the necessity to intubate the patient). Complications were not further considered for this review. Based on their symptom list, systematic reviews on the association of SARS-CoV-2/COVID-19 and the listed symptoms were screened for the probability of a causal relationship between acute SARS-CoV-2 infection and the analyzed acute otorhinolaryngological symptom [30,31,32,33,34,35,36,37,38,39,40,41,42,43,44,45]. The results of this analysis are summarized in Table 1. The classification of Tirelli and Boscolo-Rizzo was simplified for this review [12], in such a way that the otorhinolaryngological symptoms were classified only in two categories: the degree of probability of a causal relationship between an acute SARS-CoV-2 infection and the reported otorhinolaryngological symptom was classified into definitive/highly probable or possible. Definitive/highly probable means that the causal relationship can be proven or probable with high scientific evidence. This includes the fact that pathophysiological mechanisms have been detected that explain the reason for long-term or even permanent damage. Possible means that the symptoms exist in an appropriate time frame with the infection, but there is no high-level evidence that proves there is a causal relationship between the infection and symptoms. There still might be no association other than mere chance. Some of the otorhinolaryngological symptoms (like rhinorrhea, sore throat, sinonasal pain, hoarseness) are typical symptoms also seen in other viral infections of the upper respiratory tract (like SARS, influenza, common cold [46]); are typical for more severe viral infections in general (like xerostomia, stomatitis, cheilitis, glossitis); or reflect the reduced general state of health (like dizziness). Some symptoms (like fever, cough, dyspnea) can also be signs of a lower respiratory tract infection [45], i.e., are not otorhinolaryngological symptoms in a narrower sense. Other symptoms are more prominent or specific to SARS-CoV-2 infection-related symptoms (anosmia, ageusia), i.e., symptoms with much higher prevalence in SARS-CoV-2 infection than other comparable viral infections. The symptoms listed in Table 1 were used for the subsequent literature research on Post COVID-19 and Long COVID.

### 3.3. Search Strategy to Identify Studies on Post/Long COVID Otorhinolaryngological Symptoms

New scientific studies are increasingly based on the WHO case definition. However, previous studies have not always strictly adhered to the definition of Post COVID-19, taking into account the period of 12 weeks after diagnosis or the suspected onset of SARS-CoV-2 infection. In addition, some studies consider symptoms in the period between 4 and 12 weeks after the onset of infection and beyond the limit of 12 or more weeks in parallel. Therefore, Long COVID was understood in the literature research for this review to refer to the long-term health consequences of a SARS-CoV-2 infection, as this covers the entire period beyond the acute phase of the disease. If health complaints in the articles were explicitly described as extended over more than 12 weeks, strictly speaking, these should be referred to below as a Post COVID-19 condition. A sharp distinction between Post COVID-19 and Long COVID did not make sense for the literature research and conclusions of this article. Therefore, because of this potential weakness of the literature, we chose to group the two similar conditions under the term “Post/Long COVID”.

As a starting point, a careful review of current Long/Post COVID-19 clinical guidelines was conducted (Table 2). This helped ensure that the present review was focused on the most relevant otorhinolaryngological symptoms. This systematic review was conducted in three steps in accordance with the Preferred Reporting Items for Systematic Reviews and Meta-Analyses (PRISMA) guidelines [47], using the following process: We conducted a systematic literature search for publications in the English language since 2019 using the PubMed and ScienceDirect databases. The year 2019 was chosen because the COVID-19 pandemic started in this year. The following MeSH terms were used: “post COVID”, “long COVID”, “chronic COVID syndrome”, “post-acute COVID-19 syndrome“, “post-acute COVID syndrome”, “post COVID syndrome”, “post COVID condition”, “post COVID”, “long term COVID”, “post-acute sequelae of COVID-19”, “persistent”, and “persistence”. In order to not miss important articles, the terms “SARS-CoV-2” and “COVID-19” were also combined with the aforementioned terms. To focus on the topic of this review, these terms were combined with the otorhinolaryngological symptoms/signs of Post/Long COVID, as revealed beforehand (see above, Table 1): “olfaction”, “smell”, “taste”, “anosmia”, “hyposmia”, “parosmia”, “phantosmia”, “ageusia”, “hypogeusia”, “olfactory dysfunction”, “gustatory dysfunction”, ”smell dysfunction”, “taste dysfunction”, “hearing loss”, “hearing impairment”, “tinnitus”, “audio and vestibular symptom”, “sudden hearing loss”, “dizziness”, “vertigo”, “balance dysfunction”, “facial palsy”, “facial paralysis”, “hoarseness”, “dysphonia”, “voice disorder”, and “cough”. These terms were searched in combination with the general terms “ear nose throat manifestation”, “otolaryngology”, “otorhinolaryngology”, “otolaryngologic”, and “otorhinolaryngologic”. Only human studies were reviewed.

A total of 152 records were retrieved in this research. Finally, a total of 108 manuscripts were included in the present narrative review based on relevance and scientific evidence. A flow diagram of this research is presented in the Supplementary Materials (Supplementary Figure S1).

### 3.4. Consensus Strategy

The highest level of evidence was found in the systematic reviews and meta-analyses (Oxford Centre for Evidence-based Medicine level 1a). Randomized controlled trials were rare (level 1b). Most other studies were retrospective, observational cohort studies (Oxford Centre for Evidence-based Medicine level 2b, 3b, 4). If possible, primarily, level 1a results were presented. If only level 2b, 3b, or 4 evidence was available, this was clearly indicated, i.e., at least considerable benefits substantiated by non-first-class evidence, according to international standards and the Association of the Scientific Medical Societies guidelines, were presented [53]. The most important otorhinolaryngological symptoms were discussed in depth, and a consensus was proposed. The manuscript was circulated among all authors until a strong consensus (100% of all authors) was reached for all statements and recommendations.

## 4. Results

The most important otorhinolaryngological symptoms reported by Post/Long COVID patients are presented. For each symptom, data on prevalence, etiopathology, and treatment are shown. For most symptoms, it was important to differentiate between symptoms reported by patients in surveys and data based on psychophysical or objective measurements. The prevalence was lower for most symptoms when relying on psychophysical or objective measurements. The results of this review concerning the estimated prevalence numbers of Post/Long COVID-associated otorhinolaryngological symptoms 6–12 months after acute SARS-CoV-2 infection are summarized in Table 3. An overview of treatment recommendations is given in Table 4.

### 4.1. Olfactory Dysfunction

*Prevalence*. It is important to note when evaluating the prevalence of long-term olfactory dysfunction that in the general population, hyposmia occurs in about 15% and anosmia in a further 4% [54]. One of the most common causes of long-term or permanent olfactory dysfunction in general is a preceding viral infection of the upper respiratory tract including infections by the coronavirus family [55]. However, two aspects are striking. First, parosmia is very common, experienced by approximately 7–28% of COVID-19-positive patients. The onset is delayed. The median onset is 2.5 months after the start of smell loss [31]. Second, the prominence of long-term smell problems after a SARS-CoV-2 infection is notable, as it is a frequent and characteristic symptom of Post/Long COVID [56]. Self-reported olfactory dysfunction is moderately reliable when felt as a sudden loss of smell during acute SARS-CoV-2 infection [57]. A more reliable method for confirming whether a patient is anosmic or hyposmic is psychophysical olfactory testing [57]. In contrast, validated psychophysical olfactory testing for parosmia is not widely used [31]. Most long-term data are published for patients infected between October 2020 (wild-type variant) and November 2022 (omicron variant). The incidence of acute smell and taste loss fell dramatically recently with the omicron variant (only about 10% of the patients reported smell or taste loss [58]). In 80–95% of patients with COVID-19-associated olfactory disorders, the ability to smell is largely restored within 1–2 months [59,93,94]. After 6 months, 96% of patients recover their sense of smell [59]. The figures from Post/Long COVID outpatient clinics must be viewed differently, as this is a selected patient population: four months after infection with the alpha variant, about 35–40% of those previously infected seen in a Post/Long COVID outpatient clinic (i.e., a selection of more severely affected cases) are normosmic, 40% are still hyposmic, and 20–25% are still anosmic [57]. The majority of hyposmic patients also complain of parosmia and about 10% of phantosmia [95]. In patients with former olfactory dysfunction seen about 2 years after acute infection again in a Post/Long COVID outpatient clinic, 25% are normosmic, 50% hyposmic, and 25% anosmic [95]. After 3 years, 2% of non-hospitalized and 5% of formerly hospitalized COVID-19 patients of a large cohort from the US Department of Veterans Affairs reported a persistent loss of smell [96]. Female sex, a greater initial severity of dysfunction, and nasal congestion are negative prognostic factors for the recovery of their sense of smell [59].

*Etiopathology*. Direct damage to the olfactory neurons seems to not be a reason for olfactory dysfunction as olfactory neurons do not express angiotensin-converting enzyme 2 (ACE2) and transmembrane serine protease 2 (TMPRSS2), key factors for SARS-CoV-2’s entry into cells [97]. In contrast, several types of supporting cells in the olfactory epithelium express these factors. The loss of supporting cells and stem cells seems to lead to thinning and a lack of repair of the olfactory epithelium, resulting in the loss of olfactory dendrites, which likely explains the prolonged olfactory dysfunction [98]. This lack of repair is accompanied by the persistent inflammation of the olfactory epithelium as is also seen in other COVID-19-affected organs [99,100].

*Treatment*. There is no specific treatment for COVID-19-associated long-term olfactory dysfunction. Evidence is insufficient to provide a recommendation for or against any intervention. Due to its simplicity and safety, consistent and structured olfactory training can be attempted [50,83]. The idea is to stimulate the regeneration of olfactory receptor neurons in the olfactory mucosa. Classically, the scents rose, lemon, eucalyptus, and clove are used here [101], whereby each of the four scents should be smelled for 30 s in the morning and in the evening over a period of weeks and months until the ability to smell has normalized again [17]. There are contradictory reports regarding treatment with systemic or topic intranasal corticosteroids [56,84]. Intranasal insulin might be an option, but more trials are needed [85,102]. Insulin has a direct effect on olfactory signaling, stimulates growth factors in the olfactory epithelium, and thus might stimulate olfactory recovery [85]. Also, other interventions should be suggested only in clinical trials [50].

### 4.2. Gustatory Dysfunction

*Prevalence.* It is very important to note that the incidence of self-reported gustatory dysfunction (about 50% of all patients) is higher than that confirmed by psychophysical gustatory testing (12–30%) during the acute phase of COVID-19 infection [60,61]. Those affected have difficulty distinguishing between an olfactory and gustatory disorder. Furthermore, one should be aware that about 5% of the general population has gustatory dysfunction [62]. If affected during acute SARS-CoV-2 infection, most persons show hypogeusia, i.e., ageusia is much rarer [63]. A population-based analysis using psychophysical gustatory testing 4 months after COVID-19 infection with the alpha variant showed that 90% of those previously infected had normogeusia, about 10% had hypogeusia, and 0% had ageusia [57]. This is in contrast to the olfactory dysfunction results, see above, where the complete loss of sensory function is much more frequent. About one year after COVID-19 infection, about 30% of prior COVID-19 patients had some degree of taste dysfunction compared to 21% in matched individuals without previous COVID-19 infection. This difference was not statistically significant [64]. At 30, 60, 90, and 180 days after the loss of taste function, the percentage of patients with persistent taste dysfunction dropped to about 21%, 12%, 10%, and 2%, respectively [59]. Those with alpha variant infections exhibited more taste loss [64]. Complete taste loss (ageusia) as a long-term complaint of a COVID-19 infection confirmed by psychophysical gustatory testing is extremely rare. During recovery from acute COVID-19 infection, the long-term taste loss perceived by many patients with Post/Long COVID likely reflects the loss of flavor sensations from odorant molecules reaching a damaged olfactory epithelium via the nasopharynx retronasally rather than the taste buds [64]. Patients may then confuse an olfactory disorder with a taste disorder when self-interpreting their chemosensory symptoms [65]. Hence, for confirmation, if a patient complains of long-term taste loss as a symptom of Post/Long COVID, psychophysical gustatory testing is mandatory.

*Etiopathology.* The etiopathology of taste loss after COVID-19 infection is not as clear as that for smell loss, but it is probably multifactorial, involving direct viral damage to taste receptors, indirect effects of olfactory dysfunction, potential neurological involvement, systemic inflammation, and the possible disruption of saliva production. It may persist for months if nerve cell damage is part of the etiology. The SARS-CoV-2 virus here uses, as in other body parts, the ACE2 receptor to enter cells. These receptors are present in the epithelial cells of the tongue and oral cavity, which include taste buds [103]. The direct infection of these cells can lead to the dysfunction or death of taste receptor cells. The immune response to the virus, including the release of cytokines, can lead to local inflammation in the oral mucosa, further damaging taste receptors [86,104]. Viral or inflammatory damage to cranial nerves (CNs), like the facial nerve (CN VII), glossopharyngeal nerve (CN IX), and vagal nerve (CN X), which are involved in taste sensation, might theoretically contribute to taste loss and central nervous system (CNS) involvement in the course of cortical taste processing [87]. Finally, salivary gland dysfunction in acute SARS-CoV-2 infection has been described. Its consequence is reduced saliva production, which is essential for dissolving taste substances and facilitating their interaction with taste receptors [105]. Critically, all mechanisms described here seem to be reversible in most cases as permanent taste dysfunction is a rare event. It is speculated that a SARS-CoV-2 infection-induced deficiency in zinc is a factor leading to long-term taste dysfunction [106].

*Treatment.* At present, fully validated treatments are still lacking for COVID-19-associated taste dysfunction [87]. Different types of treatments have been tried in patients with persistent taste dysfunction. These treatments include the use of tetracycline, corticosteroid, zinc supplementation, stellate ganglion block, phytochemical curcumin, traditional herbal medicine, nutraceutical vitamin D, and/or photobiomodulation [87]. Unfortunately, the level of evidence has been insufficient to support their routine use in clinical practice [86,87].

### 4.3. Facial Palsy

*Prevalence.* An isolated facial palsy during acute SARS-CoV-2 infection is a rare event. An incidence of about 0.1–1% of facial palsy diagnoses is reported within 2 months of COVID-19 infection [70,71]. Most studies do not make a clear difference between Bell’s palsy and facial palsy in association with SARS-CoV-2 infection. Strictly speaking, Bell’s palsy is an idiopathic facial palsy, i.e., facial palsy induced by an acute SARS-CoV-2 infection cannot be Bell’s palsy. Furthermore, it has to be taken into account that COVID-19 mRNA-vaccinated persons have a slightly increased risk for Bell’s palsy [72,73]. About one-third of cases are diagnosed with Guillain–Barre syndrome (GBS) associated with COVID-19 [34,40,74]. This is the reason why some authors categorize facial palsy during acute COVID-19 into GBS and non-GBS facial palsy. The GBS relation might be the reason why a high rate of bilateral palsies is reported (about three out of four GBS cases). But at about 12%, the reported rate of bilateral non-GBS facial palsy cases is high [40]. Most cases are unilateral with variable severity from mild to complete facial paralysis. Complete facial paralysis recovery in non-GBS patients is achieved in about two-thirds of patients within a median of 11 days [40]. This might be the reason why persisting facial palsy, incomplete recovery, or the development of facial synkinesis is not reported in any larger clinical trial on Post/Long COVID symptoms. Hence, the prevalence of such longer-term symptoms seems to be 0%.

*Etiopathology*. Cranial nerve involvement is responsible for many symptoms of COVID-19. Mainly, two mechanisms are discussed. Dissemination to the central nervous system via hematogenous spread or trans-neuronally via cranial nerves causing direct neuronal damage due to viral neurotropism is one proposed mechanism. Neuronal damage secondary to an abnormal immune-mediated response might be a second mechanism, especially in GBS-related cases [40].

*Treatment.* During the acute phase, most isolated facial palsies are treated like Bell’s palsy, i.e., treatment mainly includes the use of corticosteroids alone or in combination with antivirals [34]. But some patients also receive antibiotics or no treatment [34]. However, 80% of patients show complete recovery [34]. Whether the prognosis of non-GBS facial palsy is comparable to the outcome of Bell’s palsy is not yet clear. In practical terms, as persisting symptoms of facial palsy are not reported as a Post/Long COVID symptom, no specific treatment has to be recommended. If confronted with a case with a presumed association, the patient should be treated as they would in any other case of long-term sequelae of facial palsy [107,108].

### 4.4. Audiovestibular Symptoms: Acute Hearing Loss, Tinnitus, and Vertigo

*Prevalence.* Audiovestibular symptoms, such as hearing loss, tinnitus, and vertigo, were reported by 8–22%, 14–15%, and 7–15% of patients with acute SARS-CoV-2 infection, respectively [37,76]. It has to be emphasized that there is a clear difference between self-perceived audiovestibular symptoms in relation to an acute SARS-CoV-2 infection and audiovestibular symptoms that can be verified by hearing and balance tests [77]. Larger series analyzing patients who reported audiovestibular symptoms during COVID-19 with standard psychophysical hearing tests (for instance, pure tone audiometry) or objective measurements (for instance, otoacoustic emissions [OAEs]) are lacking. It was not possible to conduct comprehensive audiological examinations in infected and isolated patients or those admitted to the intensive care unit [78]. On the whole, case reports on acute hearing loss in connection with a COVID-19 infection and confirmation by hearing tests are published [78]. OAEs for proving inner ear damage were rarely used. If used, OAEs were not detected in most cases, but some patients also received potentially ototoxic medications. Smaller series with hearing tests report a Post/Long COVID prevalence of about 5–12% for hearing loss and 8–10% for new tinnitus [109]. Subjective tinnitus cannot be verified by objective testing. Its characteristics are highly variable concerning noise character, sensation level, and laterality [37]. About a quarter of patients with chronic tinnitus reported the exacerbation of tinnitus regardless of whether they were infected or not [79]. Up to 9–18% of patients who recovered from COVID-19 reported persisting equilibrium disorders in questionnaires after COVID-19 diagnosis [80]. Balance testing was not performed in larger series of patients complaining of acute balance dysfunction in the acute COVID-19 phase. An unselected series of COVID-19 patients showed no significant change in the hearing status at the time of diagnosis and 3 months later compared to a control group [81]. There are not enough reliable data published to present recovery rates from audiovestibular symptoms [78]. This holds true for recovery from all important symptoms, i.e., hearing loss, tinnitus, and balance problems [37]. Patients with Post/Long COVID symptoms like cognitive impairment, fatigue, or insomnia also often complain of balance dysfunction. This is related to cortical changes and/or secondary psychosomatic disorders but not to the dysfunction of the peripheral vestibular organs [82].

*Etiopathology.* There is only speculation but no sufficient scientific evidence for a connection between acute virus infection and audiovestibular symptoms. Acute hearing loss might be related to the central and/or peripheral involvement of the auditory pathways. The cause might be indirectly (e.g., thrombosis leading to ischemic damage) or directly (e.g., viral spread and neuroinflammation) related to SARS-CoV-2 [110]. It is generally accepted that a causal relationship with the occurrence of tinnitus can only be possible if inner ear damage can also be proven in the causal relationship with the acute event, here, a SARS-CoV-2 infection, at the same time. Especially in patients with severe acute SARS-CoV-2 infection who need treatment in an intensive care unit, the intake of potentially ototoxic drugs can also cause hearing loss, tinnitus, and vestibular damage. This must be distinguished from the direct effects of the infection.

*Treatment.* Most cases of acute audiovestibular symptoms were treated similarly to idiopathic sudden hearing loss or acute vestibular dysfunction, i.e., with oral, intravenous, or/and intratympanic corticosteroids. A minority also received hyperbaric oxygen therapy [78]. The rationale here is that early neuroinvasion by SARS-CoV-2 of the central nervous system, including respiratory centers, leads to brain hypoxia of the infected parts of the central nervous system [111]. However, the outcome is unclear. A specific treatment for the acute phase is not established. Tinnitus counseling should be performed as for any other case of tinnitus. Persistent hearing loss or vertigo is treated as in any other patients with these complaints [91,92].

### 4.5. Cough

*Prevalence*. Dry cough is one of the most common presenting symptoms of COVID-19, reported in about 60–70% of symptomatic patients [38]. The cough persisted for an average of 19 days in the early phase of the pandemic in 2019–2020. The cough, nevertheless, lasted for 4 weeks or more in approximately 5% of patients infected in 2020. For hospitalized patients, the number was higher, with about 18–23% reporting persistent cough 2 months after discharge [38,66]. The estimated prevalence at 12 weeks was about 7–14% [67]. When the patients in England and Wales were surveyed, 20–30% reported persistent cough 2–3 months after the onset of symptoms of COVID-19 [38,68]. In a Chinese prospective, longitudinal cohort study enrolling formerly hospitalized COVID-19 survivors who exhibited residual lung abnormalities, 12% still complained of cough 3 year after onset [69]. Here, cough was thought to be mainly related to residual lung diseases.

*Etiopathology*. As the cough reflex is mediated by the vagus nerve [112], interactions between the virus and the airway-related fibers of the vagus nerve, with ensuing neuroinflammation, represent the likely primary events for the initiation of cough. The coronavirus-binding receptors ACE2 and TMPRSS2 are markedly expressed on the cilia of respiratory epithelial cells. This is the reason why the virus causes severe respiratory diseases by infecting the cells of the upper respiratory tract, bronchial epithelium, and lungs [113]. Another explanation is that the transnasal viral invasion of the central nervous system leads to neuroinflammation and this then to the dysfunction of central networks including the autonomous regulation of respiration and the cough reflex [114]. It is hypothesized that SARS-CoV-2 infection leads to a cough hypersensitivity state in patients with persisting cough [38,115]. Post COVID-19 syndrome might also result from neuroinflammatory events in the brain [38].

*Therapy*. An evidence-based specific treatment for COVID-19-related persisting cough is not established. It is recommended to follow guidelines on the treatment of chronic cough [88]. Different treatable traits exist depending on the mechanism to be addressed. Patients may respond to anti-inflammatory treatment and non-acid reflux being treated with promotility agents rather anti-acid drugs [88]. Another strategy is to reduce hypersensitivity via neuromodulation. Low-dose morphine is also highly effective in a subset of patients resistant to other treatments [88]. Finally, the use of gabapentin and pregabalin is also reported in a small number of patients [88]. If chronic cough is related to a voice disorder (see below), speech therapy can also be indicated [89].

### 4.6. Hoarseness and Dysphonia

*Prevalence.* The prevalence of COVID-19-related dysphonia as a clinical symptom during acute COVID-19 infection is reported to be 15–39%, with a higher prevalence in female patients [30]. The prevalence of COVID-19-related dysphonia declines to about 11–26%. Approximately 20% of these dysphonic patients experience Long COVID dysphonia [30]. Self-reported dysphonia seems to be reliable. There is a strong association between endoscopic findings and self-reported dysphonia [75].

*Etiopathology*. Again, the mechanism by which the virus enters the body via surface proteins like angiotensin-converting enzyme 2 (ACE2) and transmembrane serine protease 2 (TMPRSS2) is relevant [30]. These proteins are highly expressed in the aerodigestive tract [116]. Moreover, ACE2 is highly expressed in the vocal tract [117]. It is also discussed that the primary replication of the virus in the nasal respiratory and/or the olfactory epithelia, followed by the invasion of the virus into the central nervous system, including the respiratory centers, occurs either along a transneural route, through the disruption of the blood–brain barrier, or both [111]. It is important to note that indirect effects like tracheostomy or intubation are not the key factors associated with COVID-19-related dysphonia [30]. Such iatrogenic organic factors have, in most patients, only short-term effects, as we know very well from other patients treated in intensive care units [118].

*Therapy*. There is no specific treatment for Post/Long COVID-related functional or organic dysphonia [30,90]. It is recommended to follow evidence-based clinical guidelines for the treatment of dysphonia [90].

### 4.7. Post/Long COVID Otorhinolaryngological Symptoms in Children

Severe COVID-19 was and still is less common in children than in adults [119,120]. The pooled prevalence of reported smell and taste dysfunction for children during acute COVID-19 infection in the early stages of the pandemic was about 8–24% and 4–14%, respectively [121]. The prevalence of Post/Long COVID in children is about 25% [120]. The persistent loss of smell (4–8%) and sore throat (2–3%) are the most common Post/Long COVID otorhinolaryngological symptoms [122,123]. The loss of smell and taste is often not clearly differentiated, as outlined above [123,124]. Higher age seems to be associated with a higher prevalence of such symptoms [122]. The loss of smell and taste is more frequently found in Post/Long COVID adolescents (12–17 years) than in Post/Long COVID-complaining school-age children (6–11 years) [124]. The presented prevalence data for otorhinolaryngological symptoms are mainly based on surveys. Hence, as for adults, it must be assumed that the real prevalence is lower. The current prevalence of otorhinolaryngology-related symptoms of COVID-19 in the post-pandemic era is much lower [125]. Hence, a decrease in new cases of children with Post/Long COVID otorhinolaryngological symptoms has to be expected. Treatment recommendations for children are the same as those for adults with otorhinolaryngology-related symptoms of Post/Long COVID. For instance, olfactory training with odors can also be applied to children starting from the age of 6 [126].

### 4.8. Predisposing Factors for Post/Long COVID Otorhinolaryngological Symptoms

In general, the cumulative incidence of Post/Long COVID during the first year is higher among unvaccinated persons infected with SARS-CoV-2 than that in vaccinated persons [127]. Furthermore, the risk is higher for persons infected early in the pandemic. The risk for Post/Long COVID was the highest during the pre-delta variant era, lower in the delta era, and the lowest compared to earlier eras in the omicron eras of the COVID-19 pandemic [127]. The incidence of Post/Long COVID in formerly hospitalized cases in the acute phase is reported to be much higher, with 50–70% of all inpatients reporting symptoms [3]. Hence, severe acute disease is associated with an increased risk for Long COVID symptoms [128]. A longer duration of acute infection (>30 days) is also a risk factor [129]. Women have been shown to have an estimated twofold increased risk of having Long COVID symptoms [128]. Recurrent COVID-19 infections are another important factor [1]. Other potential risk factors, including age, obesity, or higher comorbidity, have shown controversial results [50]. Specific risk factors for the development of Post/Long COVID otorhinolaryngological symptoms have not been identified. Nevertheless, the first machine learning algorithms have been published based on a small Saudi Arabian cohort (<500 COVID-19 patients) predicting the persistence of smell or taste loss with the best area under the curve (AUC) between 0.86 and 0.96 based on long lists of symptoms during acute infection [130]. The most important factors for persistent smell or taste loss were pain, fever, and dyspnea. In addition, female sex was a negative but only moderate predictor for the permanent loss of smell [130]. When looking at large Post/Long COVID cohorts, it is noticeable that otorhinolaryngological symptoms are not isolated. That means that patients with Post/Long COVID complain of fatigue, dyspnea, mental disturbances, and cardiovascular or muscular problems together with otorhinolaryngological symptoms [1]. One can therefore deduce that the occurrence of otorhinolaryngological symptoms is at least associated with other common Post/Long COVID symptoms.

## 5. Discussion

Otorhinolaryngological symptoms are frequent in Long/Post COVID-19 patients. The permanent loss of olfaction especially has a severe negative impact on the quality of life in adults and children [124,131,132]. Unfortunately, as for all other otorhinolaryngology-related Post/Long COVID symptoms, an evidence-based treatment cannot be recommended. Fortunately, it seems that the post-pandemic SARS-CoV-2 variants cause fewer otorhinolaryngological symptoms in the acute phase of the infection. Nevertheless, these symptoms were still frequently seen in the transition from the COVID-19 pandemic into endemic scenarios [132]. In general, however, the overall prevalence of such symptoms as part of Post/Long COVID will probably decrease.

The COVID-19 pandemic period since early 2020 had, in retrospect, an extremely important impact on the field of otorhinolaryngology [133]. It became clear that viral transmission was occurring via aerosols passing through the very areas where otorhinolaryngologists spent most of their time performing physical examinations: the nasal cavity, pharynx, and oral cavity [13]. Furthermore, there is evidence that the nose plays an important role as an entry point for SARS-CoV-2 to the brain. The virus enters the respiratory or olfactory epithelia of the nasal cavity and can spread from here to the brain through the olfactory or trigeminal tracts [111]. It is very probable that an aerosol spreading via the upper aerodigestive tract will again be an important factor when the next viral pandemic comes [134,135]. To better help patients with Post/Long COVID otorhinolaryngological symptoms and to be prepared for future pandemics, it remains important to develop otorhinolaryngological care pathways and treatment concepts for these symptoms.

**Figure 1:**
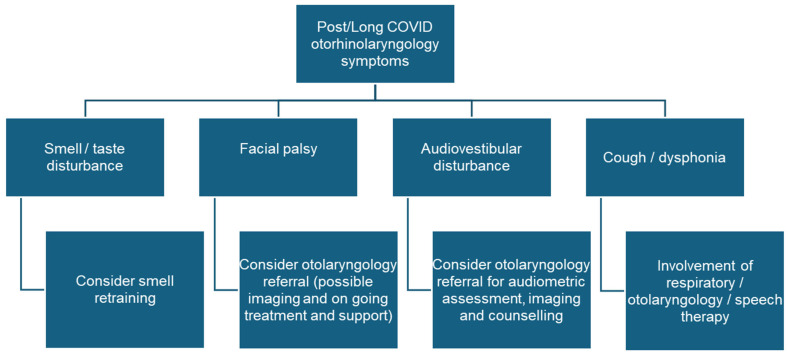
Post/Long COVID symptoms that should lead to a referral to an otorhinolaryngologist and treatment options.

Most patients visiting Post/Long COVID units present (like in the post-SARS era in 2003) with fatigue, dyspnea, mental disturbances, or cardiovascular or muscular problems [136]. In addition, we should not forget that the survivors of a formerly serious COVID-19 infection with a long stay in intensive care are only now in a condition to seek treatment for persistent symptoms. For these patients, an olfactory disorder can be of secondary importance [137]. Correspondence on the patients of these units often contains information about olfactory or gustatory disorders, although smell and taste disorders have never been confirmed by psychophysical tests [57,65]. It is therefore recommended that otorhinolaryngologists take part in Post/Long COVID unit consultations (Figure 1). Although a specific treatment cannot be offered (at least at the moment) for most complaints, this is important to confirm the diagnosis, obtain optimal counseling, and initiate a symptomatic therapy if needed.

Long COVID care is not uniform across countries and institutions [138]. Not all patients have access to specialists or Post/Long COVID units [139,140]. In general, healthcare providers seem to have increasing expertise in diagnosing and treating Post/Long COVID [137]. Primary outpatient care provided by general practitioners has much improved [141]. We could not find publications on the outpatient care of Post/Long COVID patients by otorhinolaryngologists. The care of patients with a permanent loss of smell is probably the most important. National service delivery plans are helpful, with a focus on primary care. General practitioners see more patients with the permanent loss of smell due to the pandemic. Olfactory training carried out in the community by patients themselves with appropriate structured information from general practitioners, advice, and support will be helpful. Referrals to otorhinolaryngology services in situations like red flags, specific alternative diagnoses, or failure to improve should be clearly defined and mapped out [142]. Finally, one should not forget that most of the data presented here come from high-income or middle-income countries. The working conditions for otorhinolaryngologists during the COVID-19 pandemic were very challenging, especially for those from low-income countries [143]. We could not identify any publications from low-income countries about otorhinolaryngology Post/Long COVID service.

Biomedical research has made substantial progress in identifying various pathophysiological changes and risk factors and characterizing Post/Long COVID. The disease is a multisystemic illness encompassing immune dysfunction, microbiota dysbiosis, autoimmunity and immune priming, blood clotting, endothelial abnormalities, and dysfunctional neurological signaling [3]. These pathophysiological changes are seen in common new-onset conditions like myalgic encephalomyelitis/chronic fatigue syndrome, dysautonomia, and especially postural orthostatic tachycardia syndrome [3]. Viral persistence in immune-privileged sites may trigger chronic low-grade inflammation and tissue injury and may correlate with Post/Long COVID symptomatology [2,144]. As this review showed, our pathophysiological knowledge of the development of otorhinolaryngological Post/Long COVID is often only theoretical. An international research roadmap is needed. The leading mechanistic hypotheses of the etiopathology of otorhinolaryngological Post/Long COVID symptoms presented here should be examined carefully, with the aim of guiding disease management and the development of clinical trials to test established treatments in the context of other viral diseases, existing drugs, and the development of new drugs [2].

This review and the conclusions that can be drawn from it have some limitations. Most of the cited studies on otorhinolaryngological Post/Long COVID symptoms had a retrospective design, often had small sample sizes, and might have had a lack of standardization of the assessments used. It is important to note in the studies whether the information on symptoms was based on the subjective statements of patients or was obtained using test procedures. This aspect was carefully addressed in this review.

## 6. Conclusions

Otorhinolaryngological Post/Long COVID symptoms have a high prevalence worldwide among both children and adults. The most important are persistent hyposmia and anosmia. However, evidence-based treatment options are missing. Improved healthcare policy strategies are needed to ensure that these patients are evaluated and diagnosed by otorhinolaryngologists. This would lay the foundation for enhanced patient counseling, initiate more otorhinolaryngology research in this field, and also optimize election for inclusion in clinical trials. There is an urgent need for an international call to action, encouraging more targeted research, clinical trials, and healthcare policy advancements to prevent and treat otorhinolaryngological Post/Long COVID symptoms.

## Figures and Tables

**Table 1:** Otorhinolaryngological symptoms and the probability of a causal association to an acute SARS-CoV-2 infection distinct from COVID-19 complications leading to otorhinolaryngological symptoms.

Definitive/Highly Probable	Possible	COVID Complication-Related Symptoms *
Cough **	Sudden hearing loss	Intubation injuries
Sore throat	Balance disorder/vestibular dysfunction	Periodontal disease
Nasal obstruction/congestion	Facial palsy	Drug reaction
Rhinorrhea/nasal discharge/sneezing	Tinnitus	Bruxism
Hyposmia/anosmia/parosmia		Temporomandibular disorder
Hypogeusia		Nasal swab trauma
Sinonasal pain		Acute otitis media
Hoarseness/dysphonia		
Ear fullness/earache		
Neck swelling		
Xerostomia		
Stomatitis/cheilitis/glossitis		
Dizziness/vertigo **		

* not further considered for this review; ** this is not an otorhinolaryngology-specific symptom but was presented to the otorhinolaryngologist.

**Table 2:** International and national clinical guidelines screened for Post/Long COVID-related otorhinolaryngological symptoms.

Guideline	Otorhinolaryngological Symptoms * Mentioned	Comment
Centers for Disease Control and Prevention (CDC) [48]	Problems with smell Problems with taste Cough	Otorhinolaryngological symptoms only mentioned but not addressed in diagnostic and treatment part of recommendations.
The National Institute for Clinical Excellence (NICE)/Scottish Intercollegiate Guidelines Network/Royal College of General Practitioners guidance on managing long-term effects of COVID-19 [15]	Tinnitus Earache Sore throat Dizziness Loss of taste and/or smell Nasal congestion	No specific treatment addressed.
AWMF clinical guideline Long/Post COVID [17]	Hyposmia, anosmia, parosmia, phantosmia Cough Dysphonia	Psychophysical smell tests needed as self-report is not reliable. Olfactory disorders usually show spontaneous remission and generally do not require specific treatment. If it persists for >3 months, structured olfactory training can be considered.
World Health Organization (WHO) [18,19,49]	Smell (including parosmia, phantosmia) Taste disorder	For clinical rehabilitation management of olfactory impairment in adults with post COVID-19 condition, we suggest using education and skill training for olfactory training. But no direct evidence based on effectiveness studies for rehabilitation of olfactory impairment in post COVID-19 condition is available yet.
The European Society of Clinical Microbiology and Infectious Diseases (ESCMID) [50]	Anosmia Dysgeusia Cough	According to ESCMID, after fatigue (31–58%), dyspnea (24–40%), and musculoskeletal pain (9–19%), anosmia/dysgeusia combination is (10–22%) one of most frequent Long COVID symptoms.
Australian Health Service [51]	Changes to taste Changes to smell Cough Hoarse voice	No specific treatment addressed.
Korean Society of Infectious Diseases [52]	Cough Sore throat Taste disturbance Smell disturbance	Cough >3 months: X-ray or chest CT recommended. Olfactory training can be considered.
National Academies of Sciences, Engineering, and Medicine (NASEM) [23]	Shortness of breath and cough Problems with taste Problems with smell	These symptoms or conditions can be intermittently or continuously present for at least 3 months. No specific treatment addressed.

* wording follows the terminology in the guidelines.

**Table 3:** Prevalence of Post/Long COVID-associated otorhinolaryngological symptoms 6–12 months after acute SARS-CoV-2 infection.

Symptom *	Prevalence	Comment
Anosmia	At 30, 60, 90, and 180 days: 26%, 14%, 10, and 4%, respectively [54,55,56,57,58]	Prevalence for objectified anosmia is different from that for self-reported anosmia. Self-rating of smell may correlate poorly with scores achieved on psychophysical testing. Self-rating rate is higher than tested rate. No clear prevalence difference between outpatients and hospitalized patients treated during acute COVID-19 infection, but hospitalized patients with severe COVID-19 illness are underrepresented in most population-based studies. Prevalence in prior vaccinated persons unclear.
Dysgeusia	At 30, 60, 90, and 180 days: 21%, 12%, 10%, and 2%, respectively [57,59,60,61,62,63,64,65]	Prevalence for objectified dysgeusia lower than that for self-reported dysgeusia. Ageusia is self-reported but probably extremely rarely confirmed by taste tasting. No clear prevalence difference between outpatients and hospitalized patients treated during acute COVID-19 infection. Data on dysgeusia in COVID-19 cases with prior vaccination are missing.
Cough	4–19% [38,66,67,68,69]	Symptom can be related to upper and/or lower respiratory tract problems. Long-term cough prevalence seems to be lower in patients treated as outpatients compared to hospitalized patients for acute COVID-19.
Facial palsy	0%? [40,70,71,72,73,74]	It seems that all facial palsies recover without synkinesis or other persisting symptoms as there are no (!) reports on facial palsy.
Hoarseness/dysphonia	17–20% [30,75]	Higher rate in females.
Hearing loss	8% [37,76,77,78]	High-level evidence data not available because most patients do not receive hearing tests.
Tinnitus	20% [37,76,79]	High-level evidence data not available, highly prevalent tinnitus symptoms in general population (12 to 30%).
Vertigo/dizziness	5–26% [37,76,77,80,81,82]	High-level evidence data not available because most patients do not receive balance tests.

* the maximal level of evidence of the analyzed literature: level 3, mostly level 4–5.

**Table 4:** Therapy recommendations for Post/Long COVID-associated otorhinolaryngological symptoms.

Post/Long COVID Symptom *	Therapy	Effectivity
Anosmia	Olfactory training [50,83]	Not finally proven; due to its simplicity and safety, consistent and structured olfactory training can be recommended
Anosmia	Topical corticosteroid spray [56,84]	Is not better than olfactory training alone
Anosmia	Insulin fast-dissolving film for intranasal delivery [85]	Cannot be recommended outside clinical trials, data too preliminary
Dysgeusia	No specific therapy established [86,87]	No data available
Cough	Treated as any other case of chronic cough [88,89]	No data available
Facial palsy	Treated as any other case of permanent facial palsy [34]	Not yet reported as symptom of Post/Long COVID
Hoarseness/dysphonia	Treated as any other case of permanent dysphonia [30,90]	No data available
Hearing loss	Treated as any other case of permanent hearing loss [78,81,91]	No data available
Vertigo/dizziness	Treated as any other case of chronic dizziness [92]	No data available
Tinnitus	Treated as any other case of chronic tinnitus [37,79]	No data available

* the maximal level of evidence of the analyzed literature: level 3, mostly level 4–5.

## Data Availability

Data sharing is not applicable to this article as no datasets were generated or analyzed during the current study.

## Supplementary Materials

The following supporting information can be downloaded at https://www.mdpi.com/article/10.3390/jcm14020506/s1, Figure S1: Preferred Reporting Items for Systematic Reviews and Meta-Analyses (PRISMA) flow diagram of literature selection process.
