# Supplementary material for: Post COVID-19 and Long COVID Symptoms in Otorhinolaryngology—A Narrative Review
Source: J Clin Med. 2025 Jan 14;14(2):506. doi: 10.3390/jcm14020506 (PMC11765628; doi:10.3390/jcm14020506)
Supplement: Supplementary file 1 [file jcm-14-00506-s001.zip › jcm-3331287-supplementary.pdf]

Journal of
Clinical Medicine

MDPI

Supplemental Material

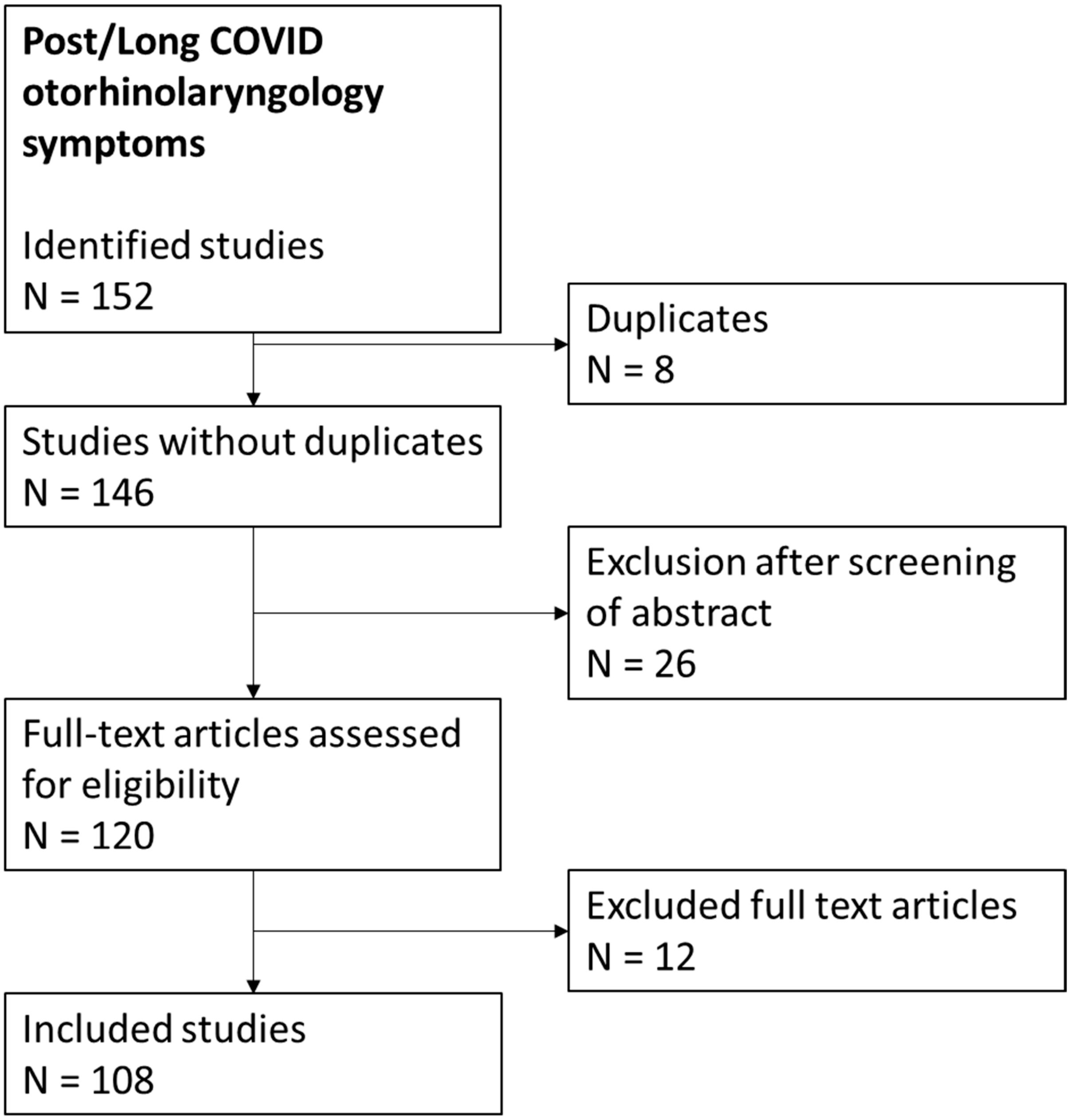

Supplemental Figure S1. Preferred reporting items for Systematic reviews and meta-analyses (PRISMA) flow diagram of the literature selection process.
